# Supplementing Public Health Inspection via Social Media

**DOI:** 10.1371/journal.pone.0152117

**Published:** 2016-03-29

**Authors:** John P. Schomberg, Oliver L. Haimson, Gillian R. Hayes, Hoda Anton-Culver

**Affiliations:** 1 Department of Epidemiology School of Medicine, University of California Irvine, Irvine, CA, United States of America; 2 Department of Informatics School of Information and Computer Science, University of California Irvine, Irvine, CA, United States of America; Centre de Physique Théorique, FRANCE

## Abstract

Foodborne illness is prevented by inspection and surveillance conducted by health departments across America. Appropriate restaurant behavior is enforced and monitored via public health inspections. However, surveillance coverage provided by state and local health departments is insufficient in preventing the rising number of foodborne illness outbreaks. To address this need for improved surveillance coverage we conducted a supplementary form of public health surveillance using social media data: Yelp.com restaurant reviews in the city of San Francisco. Yelp is a social media site where users post reviews and rate restaurants they have personally visited. Presence of keywords related to health code regulations and foodborne illness symptoms, number of restaurant reviews, number of Yelp stars, and restaurant price range were included in a model predicting a restaurant’s likelihood of health code violation measured by the assigned San Francisco public health code rating. For a list of major health code violations see (S1 Table). We built the predictive model using 71,360 Yelp reviews of restaurants in the San Francisco Bay Area. The predictive model was able to predict health code violations in 78% of the restaurants receiving serious citations in our pilot study of 440 restaurants. Training and validation data sets each pulled data from 220 restaurants in San Francisco. Keyword analysis of free text within Yelp not only improved detection of high-risk restaurants, but it also served to identify specific risk factors related to health code violation. To further validate our model we applied the model generated in our pilot study to Yelp data from 1,542 restaurants in San Francisco. The model achieved 91% sensitivity 74% specificity, area under the receiver operator curve of 98%, and positive predictive value of 29% (given a substandard health code rating prevalence of 10%). When our model was applied to restaurant reviews in New York City we achieved 74% sensitivity, 54% specificity, area under the receiver operator curve of 77%, and positive predictive value of 25% (given a prevalence of 12%). Model accuracy improved when reviews ranked highest by Yelp were utilized. Our results indicate that public health surveillance can be improved by using social media data to identify restaurants at high risk for health code violation. Additionally, using highly ranked Yelp reviews improves predictive power and limits the number of reviews needed to generate prediction. Use of this approach as an adjunct to current risk ranking of restaurants prior to inspection may enhance detection of those restaurants participating in high risk practices that may have gone previously undetected. This model represents a step forward in the integration of social media into meaningful public health interventions.

## Introduction

Food pathogens cause 9.4 million pathogen confirmed foodborne illnesses in the United States each year [1]. Each year, 38.4 million foodborne illnesses are reported, and an estimated total of 48 million foodborne illnesses (reported and unreported) occur [2]. In recent years the prevalence of certain foodborne organism contaminants such as Shigella, Vibrio, and Shiga-toxin producing Escherichia coli have been on the rise [3]. Outbreaks of foodborne illness in the United States have increased from 675 in 2009 to 852 in 2010 [4]. Restaurants have been connected to foodborne illness outbreaks in the past, however the ability to detect outbreaks is limited by variability in reporting of foodborne illness. Health departments face a burdensome task of assessing a growing number of restaurants while confronted with funding challenges. In the last year, the San Francisco, CA Department of Public Health (SFDPH) [5]conducted inspections of 7,000 restaurants and other food serving establishments one to three times a year [6][7], while the growth of the restaurant industry increased at a rate of 4.9% per year [8]. Further contributing to the problem, risk of contracting foodborne illness is also increasing on an annual basis for specific food pathogens [9]. The majority of foodborne illness cases are traced back to food served at restaurants [10]. Health code violations such as sick employees participating in food preparation and lack of employee hygiene are particularly difficult to detect given limited surveillance coverage available to local health departments [11].

Annual health inspections only capture a small window of time and may not accurately reflect the true practices of an establishment. The coverage provided by health inspection covers less than 1% of a restaurant’s annual operation time. At most, the SFDPH inspects each restaurant two to three times per year. Current risk ranking dictates that restaurants that receive a favorable health code rating (> 80 out of 100 points) will not be inspected again for the rest of the year. Restaurants with more severe findings (Major Violations) will have a greater number of points deducted from their score. Through this approach, those restaurants that have the highest risk violations will be re-inspected first. Additionally, while restaurants receiving suboptimal health department scores are more likely to be connected to foodborne illness outbreaks, suboptimal scores currently predict a minority of restaurant related outbreaks [3]. Foodborne illness can be transmitted through multiple routes, including food handler practices, contaminated food products or equipment, contamination by vermin, poor employee hygiene, or malfunctioning sanitation or food processing equipment. There are many ways that establishments may contribute to the propagation of foodborne illness, and not all of these risks can be detected by health inspectors in a short period of time [10]. Furthermore, the food production environment is very dynamic and changes with time [11,12]. Defining the utility of new epidemiologic surveillance tools that improve surveillance coverage and use alternative means of detection is a useful first step in improving and monitoring restaurant practices to achieve greater public health.

Recently, there has been an increased interest in the use of social media sites like Facebook, Twitter, Craigslist, Google, and Wikipedia to conduct different types of public health surveillance [12–30]. Using social media to enhance public health surveillance is a new approach that is beginning to gain momentum [25]. Generous et. al examined use of Wikipedia access logs to track global disease incidence and set forth four challenges to academicians that wish to identify approaches to use social media to track health measures in the real world [31]. The challenges to be met are “openness, breadth, transferability, and forecasting”. The authors argue that open source data and open source code are necessary to ensure that achievements can be built upon by third parties, and that models should demonstrate the ability to be adapted from one disease context to another. In other words, models should offer some degree of exportability. Transferability refers to the ability to use models in places where incidence data does not exist due to lack of tracking or an inability to access information. Transferable models are robust enough that they do not require new training set data when transported to new locations. Generous et al. also challenge researchers in this area to provide forecasting and “now-casting” of disease incidence, stating that “models should provide not only estimates of the current state of the world—*now-casts*—but also *forecasts* of its future state” [26]. When describing our current study, we will show how our work meets or does not meet these four challenges, and why.

Yelp.com[32] (we will refer to Yelp.com as “Yelp” from this point forward for the sake of brevity) is a social media site where individuals freely write and post reviews of restaurants and other businesses they have personally experienced. Reviewers may also assign a star rating to a restaurant that denotes the reviewer’s personal opinion of the restaurant’s quality. This star rating is also aggregated into a composite star rating that reflects the average opinion of Yelp reviewers. In addition to stars Yelp also tags each restaurant with data on how many reviews have been written on the restaurant, the aggregated “expensiveness” of the restaurant (measured by a range of 1–3 dollar signs). Reviews are scored by users according to whether they are “cool,” “funny,” or “useful.” Yelp uses a proprietary algorithm to rank the order in which reviews are viewed for any given restaurant. Yelp also applies filters on reviews to remove reviews that are not based on personal experience, incorporate spam (irrelevant hyperlinks) into their text, or are written by reviewers with few reviews on Yelp and few connections (friends) that are also Yelp reviewers.

Restaurant reviews and informative tags for restaurants can be accessed publicly through use of programming languages such as R, Python, Ruby or Perl, which are all open source [33,34]. In this way, public health officials can identify, tag, and track foodborne illness-related keywords (and other keywords) spatially and temporally in free text review fields. Such methods provide epidemiologists with key surveillance data needed to identify clusters of substandard restaurants that could alert public health officials to potential foodborne illness risks, allowing them to prioritize restaurant inspection of high risk institutions before assessing others. Such a system also allows for increased coverage of criteria that would trigger inspection, such as multiple reports of foodborne illness symptoms within a given time period, visualization of vermin, or employee hygiene breaches. This system could also be used to track prevalence of high risk restaurants in a given area and/or period of time.

However, before social media-based surveillance techniques can be employed, it is necessary to better understand and validate this method’s potential for prediction of suboptimal health inspection scores/health code violations. The first step to understanding this potential is defining the predictive power of this surveillance tool. A recent study in Mortality and Morbidity Weekly Report (MMWR) found that Yelp data in New York City could be used to identify foodborne illness outbreaks [24]. While this method of surveillance was able to identify three foodborne illness outbreaks that had gone unreported, it also required additional staff time to “read reviews and send emails” and “interview Yelp reviewers” [24]. Additional services of a foodborne illness epidemiologist were also required. This method of surveillance, while effective, may be beyond the financial means of many public health departments. It is important to note that the focus of the New York study was specifically that of outbreak surveillance. Our study focuses on surveillance of a related but different subject: surveillance of restaurant health practices. Surveillance of restaurant risk factors may add additional public health value to outbreak-based surveillance approaches. This is because surveillance of risk factors allows for interventions to be taken that may in turn prevent future outbreaks from occurring. Of course, if resources are available, then both approaches could be used in addition to traditional inspection methods.

This study validates a method of web-based surveillance similar to that conducted in New York City by the New York Health Department and the CDC [35]. This study also adds to the findings of Kang et al. [36] in identifying social media on Yelp.com as a source of data that is predictive of substandard health code rating. Our method is different from the New York study in that we aim to detect health code violations that increase risk of foodborne illness transmission. In this paper we aim specifically to present a method able to form robust predictions of health code violation prevalence, identify restaurants with high risk of health code violation, and validate increased surveillance coverage by using free text and tags created by Yelp reviewers. In contrast to Kang et al, we do not employ support vector regression to identify model terms. Rather we employ a traditional epidemiologic approach (logistic regression) to generate predictions for our model and then, provide robust validation for this approach. Our predictive model achieved these aims and was validated as a new tool that can be adopted by public health officials to improve surveillance scope and risk factor detection. Our study took advantage of the existing public health surveillance standard—the health code rating—to measure the effectiveness of our model. Furthermore, our study models prevalence of health code violation over a three year period across two different geographic regions (San Francisco and New York City). Our approach can easily be adopted by many health departments without additional expenditure in terms of time and staff. Our model specifically improves detection of restaurant risk factors, which is very different from detecting foodborne illness outbreak detection. By providing a scope of detection that represents recent observations using additional measures, inspectors may make more informed choices on which restaurants present the greatest risk and have the greatest need for re-inspection.

## Methods

The University of California Irvine Internal Review Board granted non-human subjects exemption to this study. This classification exempted this study from further University of California Irvine Internal Review Board review. The approach tested in this study is predicting health code ratings and health code violations. Health code violations are measurements of the risk a restaurant poses to public safety based on observed deficiencies during an annual health inspection. Health code violations may be cited if sick employees are working, vermin or signs of vermin are observed, sanitation equipment is used improperly, or unhygienic behavior occurs. A health code rating is assigned by a health inspector after reviewing all hazard critical control points in a food serving establishment (restaurants, food truck, sidewalk vendors). A restaurant’s health code rating is set based on the number and severity of health code violations it incurs. Restaurants are divided into three categories by SFDPH: category one restaurants are graded two to three times each year and receive scores ranging from 0–100. Category two restaurants require only one inspection a year, and category three restaurants do not require annual inspection. This study is focused solely upon prediction of scores for category one restaurants. In New York: “Each health code violation results in points; total points are a restaurant’s inspection score. Restaurants with 0 to 13 points earn A grades, 14 to 27 points earn Bs and 28 or more points get Cs. Restaurants that score in the B or C range on their initial inspection begin a new cycle of inspections sooner. Those earning 0 to 13 points on their initial inspection start a new cycle in approximately 12 months, while those scoring 14 to 27 or 28 or more points begin a new cycle in approximately six months and four months, respectively[37].” In San Francisco, higher scores indicate higher fewer health code violations, while in New York, higher scores indicate more health code violations. Our approach sets out to improve detection of restaurants that are public safety risks with little added burden in terms of time/cost. This is achieved by detecting keywords and tags via Yelp reviews that may be related to deficiencies in restaurant procedure or practice that may result in health code violation and/or citation. Reporting food poisoning or symptoms of food poisoning (along with other reported keywords) after eating at an establishment could be predictive of a variety of health code violations. However, the means of detecting this deficiency is very different from the surveillance employed by health inspectors. We employ and validate such an approach in this study.

### Coding

Coding of model terms was based upon the scoring rubric employed by San Francisco health inspectors when conducting restaurant health inspections. Keyword selection was first based on relation to hazard analysis and critical control points on which restaurants are graded. The authors then selected terms that were correlated with the low health code rating when deciding upon inclusion or exclusion in the model. Other terms in the model were directly related to aspects of the fourteen possible major health code violations a restaurant in San Francisco could receive (Please Consult Supporting Information for a complete list of major health code violations.):

Keywords and tags extracted for use in our predictive model included:

Tags: (The number of Stars awarded, The number of reviews a restaurant receives, the number of dollar signs denoting the expensiveness of the restaurant)

Keywords:”I_love”,”oldschool”,”pushy”,”pool”,”affordable”,”Christ”,”stench”,”employees”,”humid”,”septic”,”Jesus”,”hell”,”dishes”,”the_best”,”high_quality”,”adorable”,”fabulous”,”craving”,”favorite”,”excellent”,”service”,”recommend”,”professional”,”delicious”,wash_hands”,”burnt”,”ache”,”pain”,”cigarette”,”asshole”,”awful”,”rotten”,”bathroom”,”toilet”,”puke”,”fuck”,”microwaved”,”shit”,”bitch”,”sucks”,”mold”,”mice”,”spider”,”exclaim”,”filthy”,”roach”,”DIRTY”,”I_found_a”,”clean”,”diarrhea”,”vomiting”,”food_poisoning”,”dirty”,”truck”+”sick”,”stomach”+”hospital”,”fish”,”nausea”,”terrible”,”horrible”

Word extraction was case sensitive and words were extracted exactly as they appear above. Extraction of profane and or sacrilegious terms was useful as these terms reflect dissatisfaction with the restaurant employees, physical environment or food quality.

Author review of Yelp reviews across different reviewers and restaurants also proved to be useful in identifying keywords that were predictive of low health code rating. We restricted reading to only the training data of the Yelp dataset to prevent bias in model creation. Tags were first introduced into the model followed by introduction of keywords one at a time by the specific domain they represented. Domains used to organize keywords were: Reviewer Sentiment, Physical Environment, Employee Behavior, Vermin Sighting, and Foodborne Illness Symptoms.

### Data Extraction

A web extraction program was created that would parse review data and aggregated tags from the Yelp website. A full list of data fields are available on Yelp[38]. Of course, additional data collection terms can be created, such as an article’s ranking in relation to another article, or the number of times a keyword is mentioned regarding a particular restaurant. All terms listed on the Yelp academic dataset were available at the time of extraction. Full Yelp data is not required for the implementation of this approach, as only keywords and tags from the first 40 top ranked reviews were included in the algorithm (as this actually improved the fit of the model). We direct readers to consult with Yelp representatives to facilitate best practices governing data usage. Data were initially extracted from Yelp reviews on Chinese Restaurants in San Francisco. Restaurants in San Francisco were chosen due to the unique embedding of Yelp’s Local Inspector Value Entry Specification (LIVES) [25] formatted health score data in restaurant pages in the San Francisco section of the Yelp website. LIVES is a format that allows public health inspection data to be inserted into the corresponding restaurant page on the Yelp website. This allowed for review data and public health data to be extracted simultaneously. Chinese restaurants were chosen as a pilot study specifically due to their greater reported prevalence of health code violation in the SFDPH database with a prevalence of 25% in Chinese restaurants vs. 7% prevalence in other restaurants [7]. Selection of high risk populations when testing surveillance or screening tool is a standard method in conducting such cross sectional studies. By using this high-risk population we were able to validate the usefulness of our screening tool under favorable circumstances. Using this high-risk population to better measure the effectiveness of our model aided in testing model sensitivity, specificity, and area under the Receiver Operator Characteristic (ROC) curve. The ROC curve plots the true positive rate (when our model correctly predicts a restaurant will have a health score <80) against the false positive rate (when our model predicts a restaurant has a health score <80 and it does not), The discrimination threshold of the model is varied to produce the ROC Curve. To validate the generalizability of this study we followed our pilot study with a random sample that was reflective of all areas and cuisine types in the city. We parsed and analyzed extracted data using the R statistical programming language [26][27]. After extraction, pilot study restaurants were randomly separated into two datasets of 220 restaurants each. These datasets were used for training and validation of the model predicting substandard health code rating (health code rating <80). The SFDPH assigns a 1 to 100 point rating, with 1 being the least and 100 the best. This rating is later transferred to a grade scale, and scores are most heavily distributed above 60. A score below 80 corresponds to corrective action that must be taken with the restaurant. This model was then employed upon the larger sample of San Francisco restaurants that represented all areas and cuisine types in the city. This sample consisted of 1,543 San Francisco restaurants. All restaurants not classified as category 1 by the SFDPH were excluded from the sample of 1,543 restaurants and all analyses. Restaurants were randomly selected from the list of restaurants in the public dataset of restaurant inspections found at https://data.sfgov.org/Health-and-Social-Services/Restaurant-Scores/stya-26eb.

To further validate the exportability of this approach, the same model was also employed on a sample of all restaurants in New York City. This sample did not exclude any restaurants based upon restaurant cuisine type. We avoided restaurant exclusion to show that our model retains predictive power when applied to reviews describing a variety of restaurants. In order to construct our New York City dataset we extracted our tags and keywords from Yelp’s website pages for New York City. We then merged data from our sample with health score data found on the New York City Department of Health and Mental Hygiene’s Restaurant Inspection Results website. We constructed a dataset representing 755 restaurants in New York City and parsed he same keywords that we had analyzed in our pilot study. We were then able to apply the same logistic regression model that we created from Yelp reviews of Chinese restaurants in San Francisco, to data created by Yelp reviewers in New York City. This analysis allowed us to further validate the ability of our model to “now-cast” [26] the health behaviors of restaurant owners and employees in areas outside of San Francisco that may have different slang/linguistic variation, regional memes, and place different value on foodborne illness information. Furthermore, by evaluating the robustness of our model in New York we are able to display our model’s transferability in events where restaurant inspection data may not be available.

### Model Creation

The purpose of this predictive model was to classify restaurants as above or below the health code rating <80 threshold in San Francisco and >14 for New York City. The SFDPH uses this threshold to set the frequency a restaurant is inspected on an annual basis. In New York City a score >14 signifies significant deficiency and is used as a criteria for risk ranking. In New York City the health department assigns points for each violation recorded during the inspection process. Thus, high scores in New York are used to identify unsafe eating establishments, while in San Francisco low scores denote unsafe eating establishments. By using the health score threshold that each department uses to assign penalty to a restaurant we are able to apply the same model in both geographic regions. We used a logistic regression model based upon aggregated tags, keywords, and a combination of aggregated tags and keywords. Tags included: number of reviews, price (i.e., number of dollar sign symbols), Page Rank/”Usefulness” (the page a review appeared on), and number of stars (average number of stars assigned by Yelp reviewers). Keywords of positive and negative weight were also used in the model to predict a health code rating <80. Measurements of model fit can be found in (Table 1) Positively and negatively weighted keywords are displayed in (Table 2).

**Table 1:** (Model Fits across datasets). Likelihood ratio test compares models 1 and 2 only. Models 1 and 3 could not be compared due to different numbers of observations. Differences in AIC and BIC highlight different model aspects. For both AIC and BIC the lower scores are reflective of better model fit. BIC highlights tags as the driver of prediction in the model. AIC identifies the improved predictive power when using keywords with tags. LRT also identifies this relationship. Note that model fit improves dramatically when applied to data with greater degree of heterogeneity. All of San Francisco (model 4) and all of New York City (model 5) represent greater data heterogeneity than our original pilot study consisting of Chinese restaurants in San Francisco (models 1–3). This is seen when applied to datasets representing the entirety of San Francisco and the entirety of New York City.

	Akaike Information Criterion (AIC)	Bayesian Information Criterion (BIC)
	Lower scores indicate better model fit	
Model 1: Tags and Keyword	**994.079**	1284.06
Model 2: Tags	1010.34	**1029.05**
Likelihood Ratio Test (LRT) comparing models 1 and 2: *p* < 0.001**		
Model 3: Keyword	1010.34	1280.81
Model 4: Tags and Keyword (ALL SF)	159.54	390.54
Model 5: Tags and Keyword (ALL NYC)	423.40	694.56

**LRT could not be measured for SF and NYC datasets as these are other original samples that are not nested within the original dataset.

**Table 2:** An analysis of correlation between tags and keywords was used to decide which terms would be included in the model. A correlation cutoff of .05 was used for inclusion in the model unless the authors strongly believed the keyword would be useful in the model despite low correlation (e.g., high quality, food poisoning, and employees, selected for relation to food quality, foodborne illness, and employee behavior). A liberal cut off point was used to include as many predictors as possible. Correlation is specific to pilot study training data which excluded all but Chinese restaurants.

Keyword	Negative Correlation with Health Code Rating >80	Keyword	Positive Correlation with Health Code Rating >80
**Vomiting**	-0.16	Dishes	0.11
**Truck**	-0.15	Clean	0.10
**Humid**	-0.07	Recommend	0.09
**High quality**	-0.03	Excellent	0.08
**Food poisoning**	-0.03	Affordable	0.08
**Employees**	-0.02	Delicious	0.07
		Service	0.07
		Fuck	0.06
		Fish	0.06
		Favorite	0.06
		Fabulous	0.06
		Ache	0.06
		Craving	0.05
		Professional	0.05
		Pushy	0.05

Keywords were selected based upon their correlation with restaurant health code rating, and were measured by the frequency they appeared in the body of reviews related to the given restaurant. Keyword selection was also based on relation to health inspection scoring rubric and author examination of language/slang used in Yelp reviews. The most predictive models included keywords and tags combined.

As is common with language, many keywords were highly correlated. Using a robust number of keywords enhanced our model’s predictive power, however the high degree of co-linearity made it difficult to interpret the effect of specific keywords. Therefore, we used principal components analysis to address this co-linearity and combined the variability of keywords into three dimensions that explained the majority of variability in our model while at the same time enhancing model interpretation (Table 3). Dimension 1 was related to keywords describing foodborne illness symptoms (e.g., nausea, vomiting, and diarrhea). Dimension 2 was related to keywords describing vermin like mice, roach, spider, and rat, the physical environment of the restaurant (e.g., humid, smelly, clean, dirty) and the behavior of employees (e.g., rude, pushy, employee, courteous). Finally, Dimension 3 combined the overall sentiment of reviewers in regards to the restaurant (e.g., affordable, the best, I love). This approach is of particular benefit to public health officials because it would inform inspectors not only which restaurants would be likely to be “substandard,” but it would also predict the category (e.g., vermin, employee behavior, foodborne illness, physical environment, lack of positive sentiment) of observations that were powering the prediction. This information could cue inspectors to what to look for on future inspections. Please see (Table 3) for confidence intervals and odds ratios of substandard restaurant health rating for all three dimensions of covariates.

**Table 3:** Principal components analysis dimensions were set as covariates in a logistic regression model to show the predictive effect of each dimension on the outcome of receiving a health score <80. The confidence intervals show that as keywords add weight to dimensions those dimensions are associated with corresponding increase or decrease in odds of low health score within the stated confidence interval.

	Estimate	Confidence Interval 2.50%	Confidence Interval 97.5%
Reviewer Sentiment Towards Restaurant	0.90	0.82	0.97
Physical Environment and Vermin	0.97	0.84	1.0
Foodborne Illness Related Symptoms	1.12	0.99	1.2

**Fig 1:**
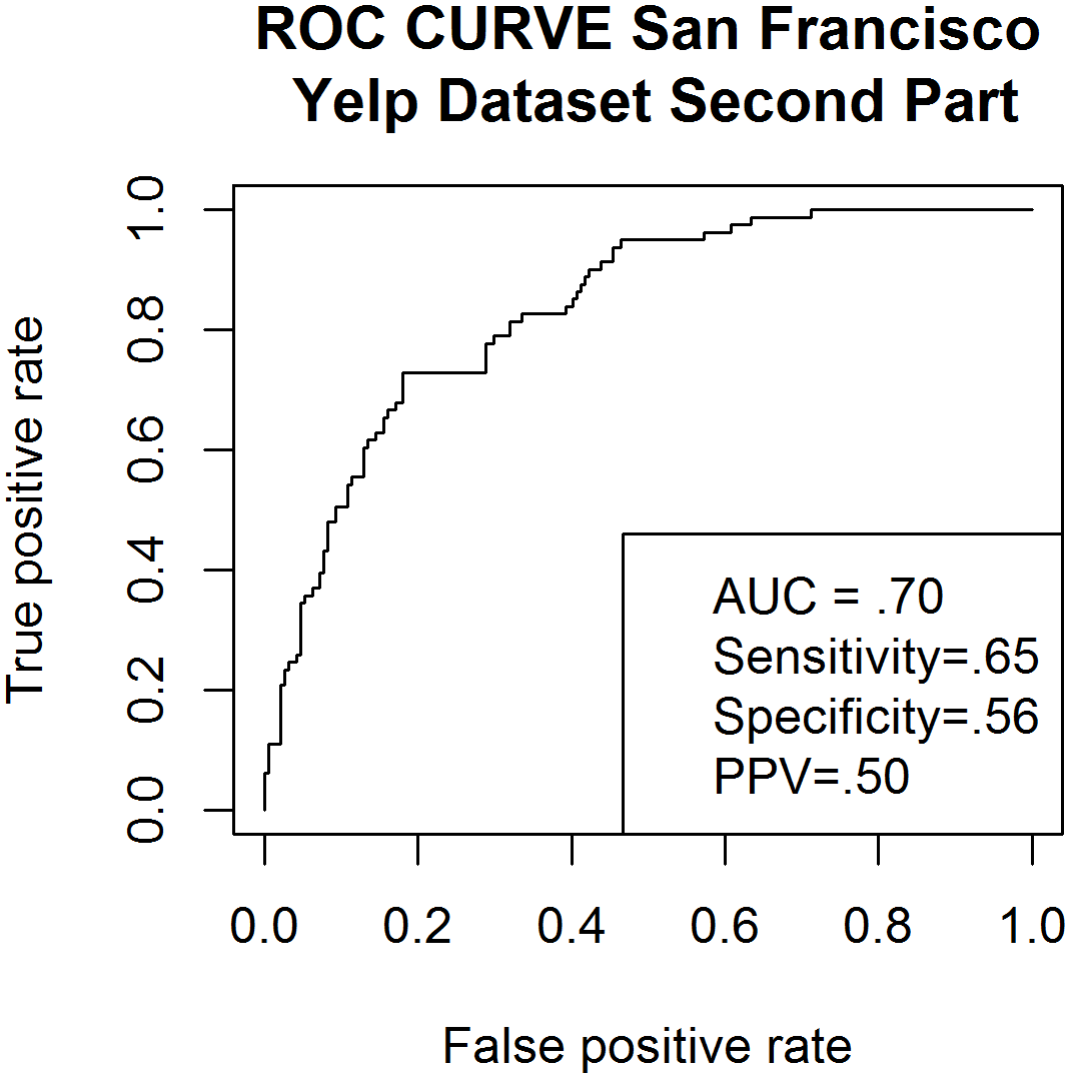
Receiver Operator Curve using validation dataset created using Yelp data compiled from 220 San Francisco restaurants.

**Fig 2:**
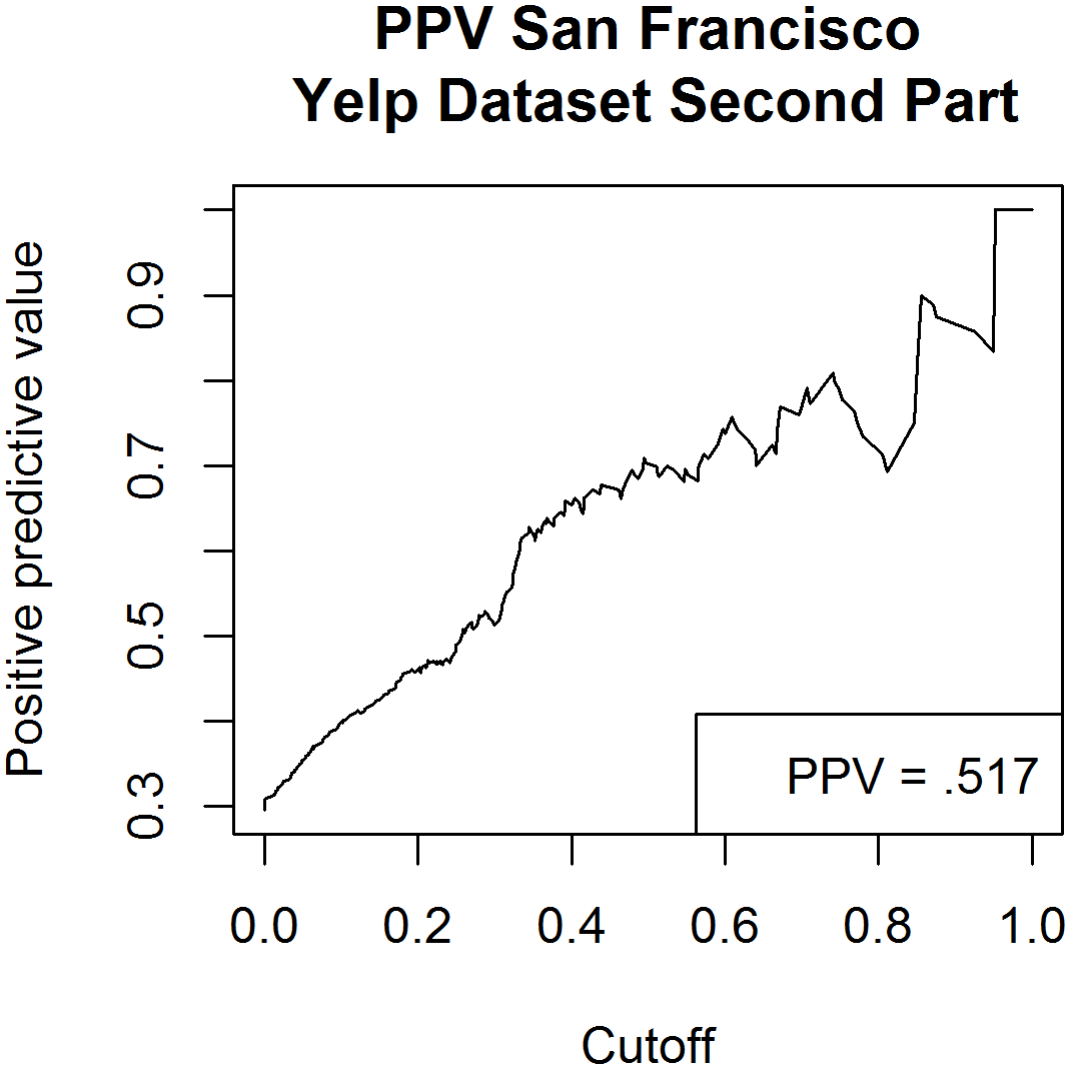
Positive Predictive Value using validation dataset created using Yelp data compiled from 220 San Francisco restaurants.

The first 220 restaurants in the pilot dataset (which excluded all restaurants but Chinese restaurants in San Francisco) were used for model training. The second part of the dataset, also consisting of 220 Chinese restaurants, serves to validate the final model sensitivity, specificity and positive predictive value. An ROC Curve was constructed to allow analysis of the area under the ROC curve/predictive power of the model (see Fig 1). ROC analysis provides a measure of the probability that our model will rank a restaurant with substandard health rating below a restaurant with adequate health rating. For San Francisco data, a health code rating <80 was the binary outcome variable being predicted by our model. This cut-off was used because it is the threshold at which the SFDPH increases the number of annual inspections from 1–2 to 2–3 inspections per year. For New York City, the scale and direction of health code ratings differ from that of San Francisco [6], thus our cutoff point also differs. New York City health code ratings included in our study ranged from 1–100, and we used a score >14 as the cut off because this score is used by health officials to categorize a restaurant as having food safety compliance below the top level. We plotted the positive predictive value using our validation dataset, San Francisco, and New York City datasets to visualize the effect of different thresholds on identifying restaurants with high numbers of health code violations (see Fig 2).

Yelp uses a proprietary algorithm to order reviews. The more “useful” a review is (according to the proprietary Yelp algorithm), the higher that review will be placed among other reviews for the restaurant. This means the most “useful” reviews will be placed on the first page with less “useful” reviews being placed on the following pages. It is important to note that Yelp users have the ability to comment on usefulness of reviews. It is possible that the user rating of “usefulness” is used for Yelp’s proprietary review ranking algorithm. If this is the case, and if the majority of Yelp users believed that reviews discussing risks for foodborne illness were important or useful, then those reviews would gain better rank than others. Under this assumption, if only the “best” reviews were used for model creation, then the model would become better at predicting restaurant health code rating. Our model’s high predictive power indicates that this might be the case. We divided groups of reviews according to which page they appeared on as a means of ordering reviews by “usefulness” as determined by the proprietary Yelp algorithm. We did this in an attempt to address whether our predictive model performed better using only reviews that were placed on the first page of reviews by Yelp. Including the usefulness term in the model allowed us to test the power of Yelp’s proprietary algorithm without needing to know how it was constructed. Inclusion of “usefulness” in our model also allowed us to measure the importance that Yelp users placed on reviews that contained keywords correlated with negative health rating, assuming that user review rating strongly influences the ranking algorithm.

As review ranking is a proprietary ranking algorithm, we should not expect its inner workings to be revealed. However, we should not consider this lack of transparency a violation of the “openness” challenge set forth by Generous et al. [30]. Yelp provides several metrics to measure the usefulness of a reviewer and a review. For a reviewer those metrics are the number of reviews they have written and the number of friends they have within the Yelp network. Metrics for a given review are how funny, useful, and cool the review is. Currently there is a clear correlation between the review metrics and the ranking of a review, and between reviewer metrics and the ranking of a review. Reviews can be pushed up in ranking by both the trustworthiness of a reviewer measured by the number of times they have written reviews, the number of people who have extended friendship to those reviewers, and the number of people who vote a review is cool, useful, or funny. As long as these metrics remain in place, an agency can assess their positive distribution across Yelp rankings in order to assess the correlation between review and reviewer metrics and review ranking. In this way even private entities like Yelp offer a measure of transparency that is sufficient when the metric/ranking relationship is measured across thousands of samples.

**Fig 3:**
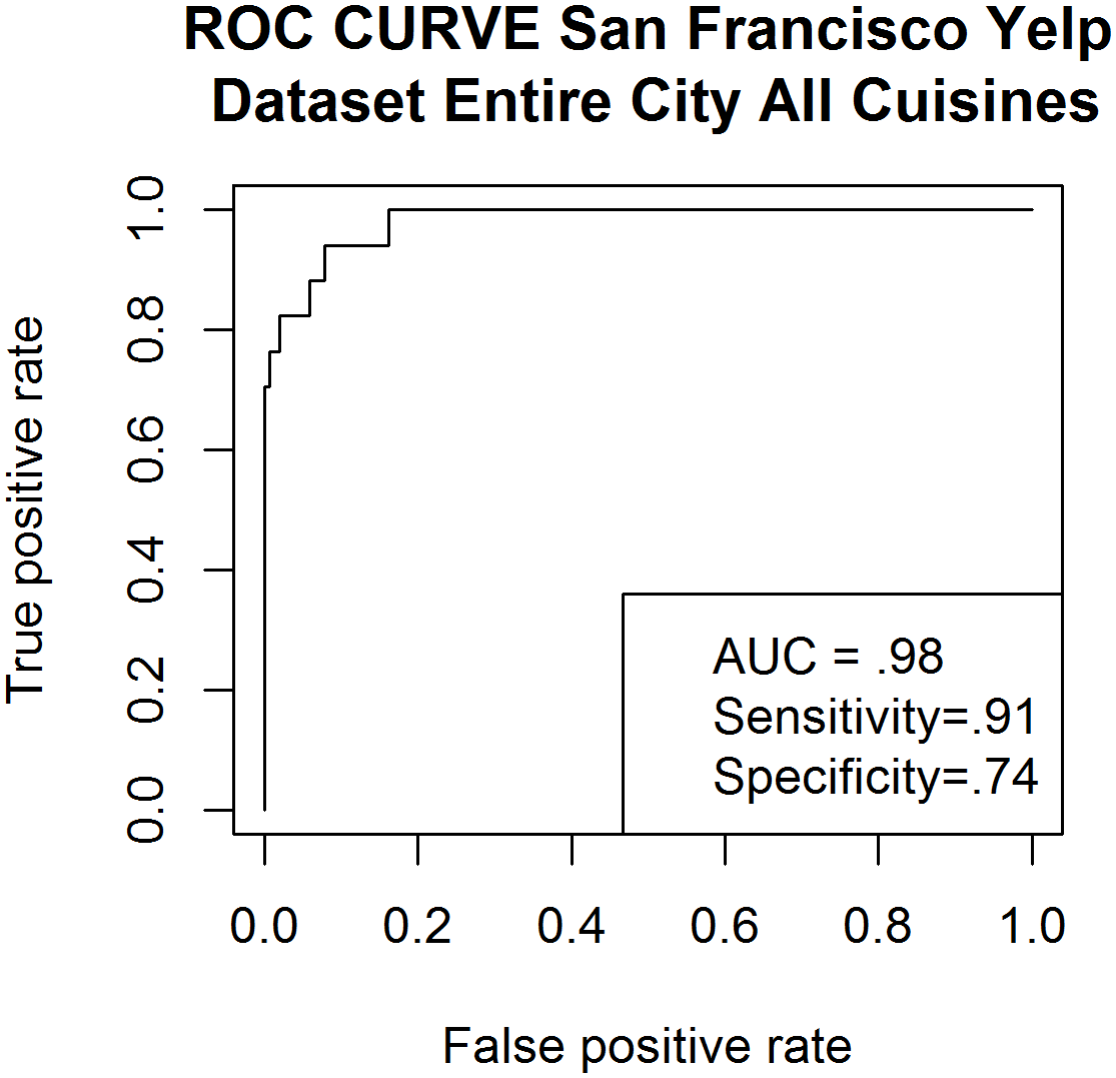
Receiver Operator Curve created using Yelp data compiled from 1,543 San Francisco restaurants including all cuisine types.

**Fig 4:**
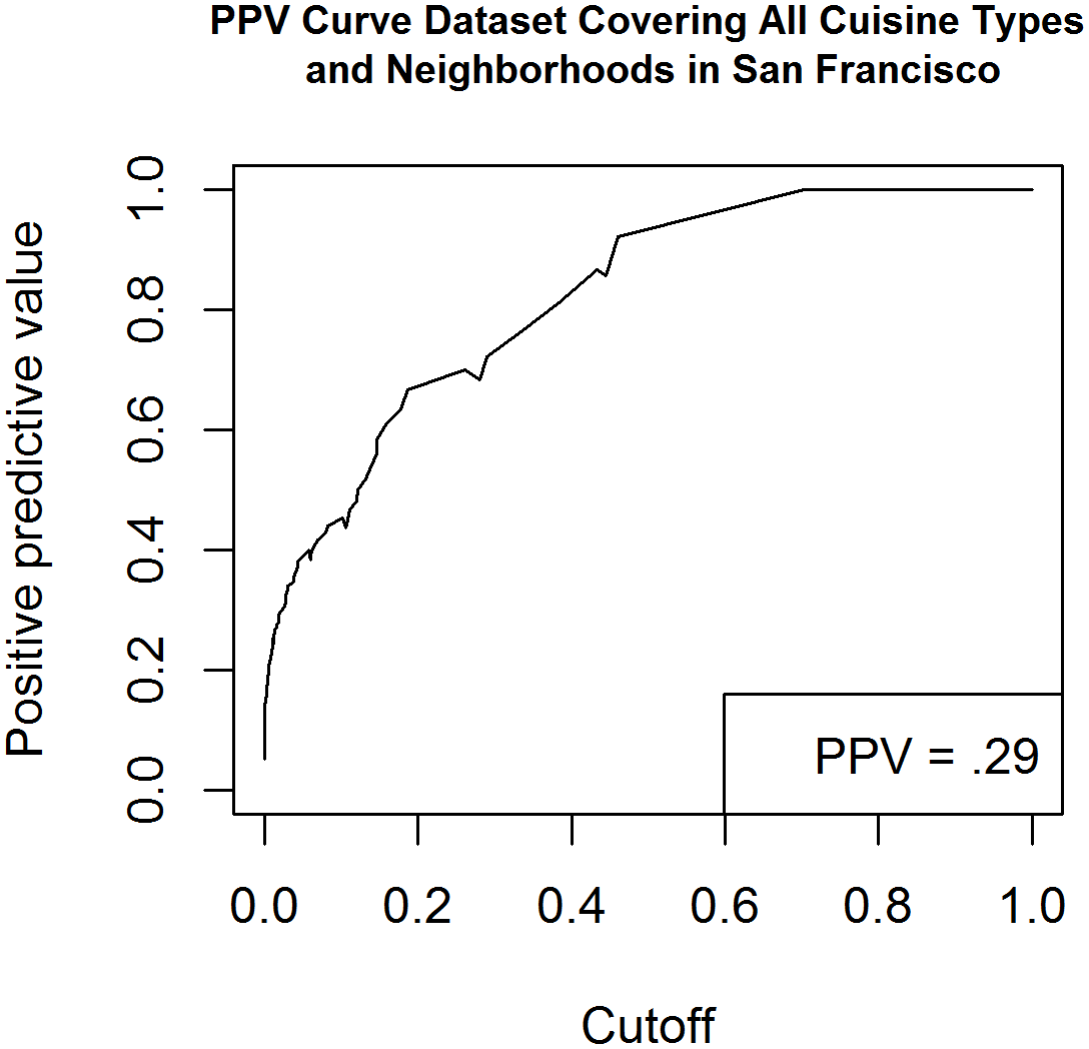
Receiver Operator Curve created using Yelp data compiled from 745 New York City restaurants including all cuisine types.

Using predicted classifications generated by our logistic regression model, we were able to compare the prevalence of low health code rating between real world observations and the predictive model. This was done to evaluate whether our model’s prediction of prevalence closely matched real world prevalence values generated by SFDPH. This part of the study was done one year after initial data collection using SFDPH data collected across inspection dates in 2014. Inspections were summed across months to more easily visualize the correlation between real and predicted values. Review data was not collected past the last day of 2014 (12/31/2014) for analysis of SFDPH data. We did not set out to determine temporality of review data; instead, the specific aim of this part of the study was to further validate the fit of the predictive model and its reliability in identifying incidence rates of substandard restaurant scores across time. We also measured the exportability of this approach by applying it to public health data from the New York City Department of Public Health. (Figs 3 and 4).

## Simulation Methods

In order to further validate our model we used bootstrap simulation to better define the predictive power in terms of positive predictive value and area under the receiver operator characteristic curve (AUC). Simulation of AUC and positive predictive value (PPV) were used because they provide a useful measure of the possible variation in our prediction metrics. The PPV is the number of true positives (when our model accurately predicts a restaurant health code rating to be <80) divided by the total number of “positives” in the dataset (the number of times a restaurant in our sample was assigned a health score <80). All simulation was conducted across 10,000 iterations of the dataset using a 200 restaurant sample size. AUC was measured across different simulated parameters such as sample size, and page rank/”usefulness”. Page rank was defined as the page number a review appeared on for a particular restaurant. If a restaurant had three pages of reviews then reviews occurring on the first page would have a page rank of one while reviews occurring on the second page would have a page rank of two and so on. For a graph of the simulated AUC across page rank please consult (S1 Fig). First page reviews were of interest because Yelp appears to order their reviews in part by user rating. Our simulation allowed us to measure whether Yelp’s proprietary algorithm for ranking reviews acted as a filter collecting more predictive reviews towards the beginning of a restaurant’s listed reviews.

To test the power of page rank/”usefulness,” we ran a 10,000 iteration loop analysis on the area under the ROC curve on random samples of the data using a sample size equal to 200 restaurants extracted from al restaurants observed. A logistic regression model was created inside each loop of the simulation. This model running on simulated data would then yield sensitivity, specificity, positive predictive value, and AUC using the R “ROCR”[39] module for each iteration of the dataset [28]. Collected predictive measures were plotted via curve, histogram, and scatter plot to visualize the predictive power across simulated sample sizes.

We then examined simulated AUC and PPV at different levels of page rank by combining both parts of the real dataset and simulating a “page rank/usefulness” spectrum where a dataset transitions from 100% “high page rank data” to 100% low page rank data (reviews occurring on first page). This was done by randomly sampling from two datasets at different proportions within a loop, and combining the two proportions at each iteration to make one dataset for which AUC was calculated. The proportion of “low page rank” reviews changed from .5% low page rank reviews to 100% low page rank reviews creating a “Yelp Page Rank/Usefulness Index”. This Index allowed us to visualize the effect of page rank/page number on the predictive power of specific reviews.

## Results

Keywords variables in our model indicated whether or not a keyword appeared at least once within a review. Keywords and tags that were not significant predictors of health code violations were left in the model if they added to the model’s ability to predict low health code rating for restaurants within the study. The average number of stars assigned to a restaurant and five keywords were significant predictors of low health code rating. For measurement of odds ratios measuring the increased or decreased odds associated with given tags and keywords in San Francisco and New York City please see Tables (4) and (5) respectively.

**Table 4:** (Significant Predictors in San Francisco Model). Odds Ratio and 95% Confidence Interval of Odds Ratio are listed above for predictive keywords and tag in the San Francisco model. Table is limited to significant predictors. Additional terms that were highly predictive but not identified as significant due to collinearity are not listed in this table.

Variable Name	Odds Ratio	Confidence Interval 2.5%	Confidence Interval 97.5%
STAR (tag)	0.64	0.43	0.96
I love (keyword)	0.05	0.00	0.42
Affordable (keyword)	0.1	0.02	0.36
Microwave (keyword)	0.08	0.01	0.79
Vomit (keyword)	45.4	1.34	273.00
Dirty (keyword)	2.21	1.43	3.68

**Table 5:** (Significant Predictors in New York City Model). Odds Ratio and 95% Confidence Interval of Odds Ratio are listed above for predictive keywords in the New York City model. Table is limited to significant predictors. Additional terms that were highly predictive but not identified as significant due to collinearity are not listed in this table.

Variable Name	Odds Ratio	Confidence Interval 2.5%	Confidence Interval 97.5%
recommend (keyword)	0.67	0.45	0.94
I found a (keyword)	7.72	1.16	45.09

We evaluated the logistic regression model in terms of sensitivity, AUC, and PPV. In the simulated datasets, the interval of AUC went from .5 to 1 as the number of restaurants using only first page reviews ranged from 0.5%-25% of restaurants. While the range of AUC narrowed to 0.75–0.9 when first page reviews were included in 75%-100% restaurants (see Fig 1). We also measured the effect of sample size on Prediction Model AUC simulating sample sizes randomly drawn from our original sample, and found that there was no increase in AUC after a 300 restaurant sample size was reached. Sample sizes were simulated from 1–1,000 restaurants.

The AUC was .79 for the first (training) part of the dataset and .7 for the second (validation) part of the dataset (see Fig 1). This means that our model accurately separated the poorly rated restaurants from the highly rated restaurants 70% of the time. The simulated dataset had a mean of .78 AUC. Thus, across 10,000 simulations our model was able to discern which restaurant would be poorly rated in 78% of restaurants on average. Measurement of model prediction effectiveness can be seen below. Simulated data were generated using 200 restaurant samples each represented by a minimum of 40 reviews. No page rank restrictions were placed on these data (Table 6).

**Table 6:** Training Data refers to the first part of the dataset used for model creation. Validation Data refers to the second part of the dataset used for validation purposes. Simulated data is a 200 restaurant random sampling and analysis repeated over 10,000 iterations using the complete dataset from the pilot study. “Prevalence” refers to the prevalence of restaurants with low health code rating in the specific dataset.

	AUC	Sensitivity	Specificity	PPV	Prevalence
Training data	0.79	0.70	0.58	0.50	0.25
Validation data	0.70	0.65	0.56	0.50	0.33
Simulated data (10,000 iterations)	0.78	0.72	0.44	0.61	0.29
Sample of all San Francisco restaurants with no cuisine exclusion	0.98	0.91	0.74	0.29	0.10
Sample of all New York City restaurants with no cuisine exclusion	0.77	0.74	0.54	0.25	0.12

The prevalence of low health code rating for restaurants on average in San Francisco is roughly 7% [7] while the prevalence of low health code rating for Chinese restaurants is approximately 25% (the prevalence of low health code rating in our pilot study sample). The positive predictive value (averaged over simulations) was 0.61 with a standard deviation of ±0.052. This indicates that the model improves the identification of restaurants with low health code rating with a 36% greater probability than chance alone.

Using bootstrap simulation our direct measurement of PPV was .5 for both parts of the simulated dataset and 0.61 for the simulated dataset (see Fig 2). The prevalence observed within our sample which was biased towards a high occurrence of substandard health code ratings (HCR <80 occurred between 26–33% of restaurants across 1^st^ and 2^nd^ parts of the dataset).

The dataset representative of the entirety of areas and cuisine types in San Francisco produced had a PPV of .29 which was nearly three times higher than the prevalence of low health code rating in our sample which was .10. Sensitivity for this sample was very high at 91% and a specificity of 71% yielding an AUC of 98% (see Fig 3). This means our model was able to rank and differentiate between adequately and inadequately performing restaurant in our sample 98% of the time Thus, of the 1,523 restaurants in our sample of all restaurants in San Francisco, our model was able to correctly classify if a restaurant health score would be <80 (substandard) in 1,493 restaurants. The dataset representative of the entirety of areas and cuisine types in New York City produced a PPV of 0.25, which is over double the likelihood of detecting a substandard restaurant by chance alone. The sensitivity of the model in detecting substandard restaurants in New York City was 74% and specificity was 54% yielding an AUC of 77% (see Fig 4). This meant that using the sample representative of New York City Yelp reviews our model (derived from a pilot study using only Chinese restaurants in San Francisco) was able to rank and differentiate between adequately and inadequately performing restaurants 77% of the time. Thus, of the 770 restaurants in our sample of all restaurants in New York City, our model was able to correctly classify if a restaurant health score would be >14 (substandard) in 581 restaurants. Calculation of sensitivity and specificity required setting a specific threshold value. To calculate a threshold value, we took the average of all scores <80 in San Francisco and found the proportion of restaurants with a lower score (10.8%). For New York City we found the average of all scores that were above a score of 14 and then calculated the proportion of scores that exceeded the average (11.7%).

**Fig 5:**
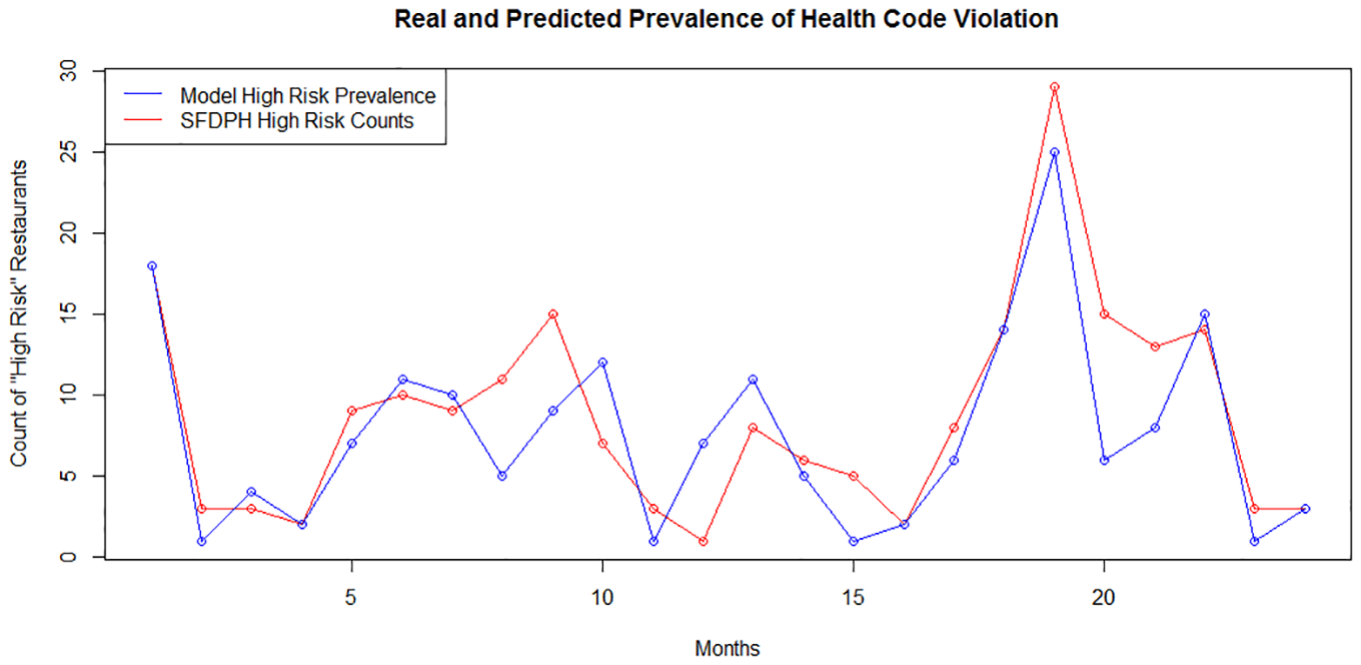
Plot of observed and predicted prevalence of low health code rating over a two year period from the beginning of 2013 to the end of 2014 using validation dataset for first year and for second year. Blue lines reflect predicted counts and red lines reflect the observed counts of restaurants with health code rating <80 (substandard).

**Fig 6:**
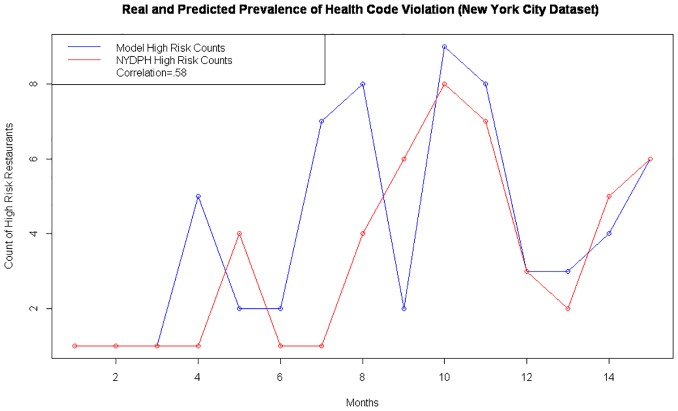
Plot of observed and predicted prevalence of low health code rating over a fifteen month period from the beginning of 2014 to the end of 2015 using sample from all restaurants in New York City. Blue lines reflect predicted counts and red lines reflect the observed counts of restaurants with health code rating >14 (substandard).

By collapsing predictions and real reports of low health rating across months we were able to plot the predicted and real prevalence of health code violations across a two year period (Fig 5). The predictive model did predict a greater than observed prevalence in 30% of the months observed, a perfect match in 25% of the months observed, and predicted less than the reported cases in 45% of the months observed. The Pearson R^2^ showed that predicted and observed values were correlated at 0.759%. A Hosmer-Lemeshow (HL) test was used to identify goodness of fit of the model. The Pearson chi squared statistic produced by this HL test was 12.39 with a p-value of 0.259 providing no evidence of lack of fit. The Hosmer-Lemeshow test was used to account for the >10 covariates included in the predicted model. Measurement of goodness of fit along with analysis of predictive power via AUC for the validation dataset of the pilot study, the boot strap simulation set, the dataset representing the entirety of San Francisco, and the dataset representing the entirety of New York City (Fig 6) validated the robustness and exportability of this predictive model.

## Discussion

There were several keywords that were significant predictors of health code violation. Unsurprisingly, aggregated Yelp “stars” are significant predictors of low health code rating. For each additional star added to a restaurant’s average star rating, the risk of low health code rating drops by approximately 36%. It is logical to assume that people who submit Yelp reviews who observe vermin, poor hygiene, or become sick after eating at an establishment are less likely to award stars than those who do not observe or experience these unpleasant things. “Affordable” and “microwave” were keywords that were significantly associated with decreased odds of low health code rating. Restaurants described as serving microwaveable products are most likely different types of food serving establishments with little to no prepping/food contact and therefore at lower risk for violation. Yelp reviewers that use a positive word like “affordable” have a good impression of the restaurant. Yelpers that perceive substandard hygiene, vermin, or those who become sick will not remark upon the affordability of the establishment. Those reviews that do remark upon negative circumstances tend to focus upon such information. In this context, there is a case why “affordable” could be predictive of an acceptable Health Code rating.

Negative keywords also predicted low health code ratings: the keyword “dirty” increased odds of low health code rating by 2.21 times on average and “vomiting” increased odds of low health code rating by 45.4 times. The keyword “vomiting” is unique in that it is very significantly associated with low health code rating. Although this keyword occurred with low frequency, we see that restaurants with low health code rating have much greater likelihood of having reviews with the keyword “vomit”/”vomiting” than those without. This shows that single keywords can be powerful predictors of low health code rating. It is important to note that words like “pain,” “diarrhea,” “poisoning” and “ache” were highly correlated with the word “vomiting.” This multicollinearity (>.7) masked the significance of these words; however these terms are useful in that they do improve the accuracy of our predictive model.

Using sample data collected from New York City Yelp reviews, our model found different keywords that were predictive of substandard health code rating (Table 6). These keywords were the keyword string “I found a” and the keyword “recommend”. The keyword string “I found a” was used to detect statements related to signs of vermin in restaurants, although the string of keywords could also detect lapses in employee hygiene (e.g., “I found a hair in my soup”). Given that this keyword string was a positive predictor of a substandard health code rating it would appear that the keyword was used to denote something negative related to a restaurant the majority of the time. The keyword “recommend” was also a significant predictor of substandard rating in New York Yelp reviews. This makes sense in the context that those reviewers that recommend a restaurant are unlikely to make negative observations related to foodborne illness, vermin, employee hygiene, or other elements pertinent to food safety. In this case, a recommendation by a reviewer adds to the model’s ability to identify restaurants that will not receive substandard rating. It should not be surprising that the keywords identified as significant in New York and San Francisco are different. This could highlight different uses of descriptive keywords in the populations of New York and San Francisco. It could be that New Yorkers are using different words to describe gastrointestinal distress they experience with food poisoning that they attribute to a certain restaurant. This system serves as an alert to inspectors to identify problems early on.

Through simulation of datasets with different proportions of reviews with first page rank we can infer that the first page of review data by itself does the best job of predicting low health code rating. As additional pages of higher page rank reviews are added, the predictive power of the model decreases. Unfortunately, because Yelp’s review rank algorithm is proprietary, we are unable to peer inside the black box to unveil why improved ranking of reviews improves their predictive power. We do know that Yelp allows users to click a box on a review if they believe it is “funny,” “cool,” or “useful.” It is possible that this user data is incorporated into Yelp’s review ranking algorithm. If so this would mean citizens of San Francisco who use Yelp may place high importance on reviews discussing keywords related to health code violations/health code rating. It is unknown if this importance would be similarly designated across other Yelp users in different geographic areas. If public health departments adopt a surveillance strategy based on user-generated content, it is important that models account for page rank to decrease the time for generating predictions.

By applying our predictive model to datasets representing the entirety of San Francisco and New York City (in addition to validation datasets from our pilot study), we have validated that this specific model will work in a variety of geographic areas. Variations in dialect, slang, and local memes may have altered the effectiveness of this predictive model. However, despite these variations our model was able to retain the power to discern substandard restaurants from their compliant counterparts 77% of the time in New York City. It is possible that speech used in Yelp reviews may not be similar to that found in other review sites, making this model only applicable to Yelp reviews. Public health workers may wish to become familiar with local Yelp speech/slang so they may populate the model with relevant keywords. In this way, area expertise can help to create a model specific to a linguistically unique geographic area. It should be noted that while this approach may increase localization, it might also make a model short lived since social memes and some slang tend to be passing fads and their usage fades. If localized models use these short-lived slang and memes, they may need to be retrained more often. Though we must also recognize that customization of models to include keyword terms that reflect local slang and social memes may further enhance the model’s predictive power. Balancing a model’s adherence to local slang and production of robust predictions over time must be addressed by public health officials and data scientists at a local level. In locales where there is an inability to provide such customization, our model will still be a useful adjunct to restaurant surveillance and can be used to rank a restaurant’s risk of violating health code regulations and possibly transmitting pathogens causing foodborne illness. In this context we see that the variability of keyword assignment that could be seen as a weakness is in fact a strength of this model in that it allows for (but does not require) increased localization of the predictive model.

A limitation of this approach is that it relies upon high participation by reviewers in the production of Yelp reviews. Yelp participation is highest in large urban areas like San Francisco and New York City, thus it should not be surprising that our model works well in these areas. As cities continue to grow and social media becomes more embedded in everyday use, the utility of this method will be likely to increase. From August 2013 to August 2015 Yelp.com experienced an increase of 4.5 million unique visitors to 6 million unique visitors on a daily basis[40]. It is also important to note that the majority of Chinese restaurants studied were centered in the cultural enclave of “Chinatown;” this is important in that it reflects that even in small geographic areas, Yelp can be a powerful screening tool if there is support from a user base within the local community.

Use of the methods outlined in this study can act as blueprint for those agencies that may wish to take the first step towards using crowdsourced surveillance. Our study used open source programming languages R and Python to extract data from Yelp using a single user account and operating within the terms of use of that account. Since ownership of a Yelp account is a good that is publicly available we feel that both the data and programming languages of this study meet the “Openness” challenge set forth by Generous and colleagues [26]. The code used for this project can easily be built upon by “third parties” to create even more robust models that may apply to other public health measures.

While our model focused specifically upon the measure of health code rating, it is possible that this model could be used for more specific health code violations that are represented by health code rating. For instance, vermin infestation or employee hygiene citations could also be detected by this model. It is likely that increasing model precision would require further localization of the terms within the model. However, the potential for this type of surveillance to be used for public health measures beyond restaurant health code rating does show that this type of surveillance could be shown to meet the “breadth” challenge set forth by Generous and colleagues [26].

We do show that this model meets the “transferability” challenge [26] in that it was shown to be robust when applied to samples generated from the entirety of restaurants in San Francisco and the entirety of restaurants in New York City. Furthermore, we demonstrated that when a model has the benefit of localization (as with San Francisco), the model can achieve a high degree of predictive power as we achieved when our model yielded an AUC of 98% when applied to the heterogeneous sample extracted from all restaurants in San Francisco.

For this study, which focused on the prediction of public health behaviors practiced by restaurant owners and employees, it does not necessarily make sense to forecast the behaviors of restaurants in the future. Instead the value of this model is that by extracting observations made by Yelp reviewers that are weighted towards the present day, we are able to “now-cast” behaviors of restaurants outside of the normal window of inspection and/or re-inspection. This increased coverage is most meaningful if the model could be used in adjunct when assigning risk to restaurants and using that risk to rank restaurants for re-inspection.

Future studies including restaurants in multiple geographic areas or restaurants representing cultural enclaves would be warranted to further validate the findings we report here. However, our sole purpose was to define the effectiveness of this surveillance approach, and a small specifically-defined population and our larger follow-up studies were ideal for defining this approach’s effectiveness. This study takes a step forward in identifying how social media data can have applications for public health departments in metropolitan cities.

Our findings offer a first step towards the meaningful use of social media data in public health interventions. It is important to note that the SFDPH is currently participating in Yelp’s local inspector value entry system (LIVES) formatting system[41]. This means that individuals writing reviews on Yelp will also have access to health code rating information on Yelp. This system, however, remains unproven, and usage statistics of public health data on Yelp are unknown. Health code violation data on Yelp can only be accessed through hyperlinks that are not easily identified, and health code ratings are on the periphery of Yelp restaurant pages and may be difficult for users to detect. Without an evaluation of this new feature offered by Yelp we are unable to measure any inherent bias that it may create in our model. Furthermore, we tested this model in New York City, which has not yet introduced Yelp LIVES formatting, and we still identified a strong predictive power of our model.

Misattribution of foodborne illness symptoms is a common finding in foodborne illness outbreak investigation. We cannot say with certainty that all keywords related to foodborne illness used in restaurant reviews represent foodborne illness that is causally linked with the specific restaurant for which the review was written. However, if we identify a high frequency of keywords related to foodborne illness (along with other predictive keywords and tags) in the body of reviews for a given restaurant, then our results indicate that restaurant would have a 45 times greater odds on average of receiving health code violation/substandard health code rating. We observed this effect in samples generated from restaurant review and tags found in two large municipalities (New York City and San Francisco). Although we cannot expect all cities to have populations of Yelp reviewers and users that rival New York City and San Francisco today, as urban areas becomes denser and participation in social media becomes more common, we expect the predictive power of this approach to continue to grow.

This study offers proof that there is validity in using social media data to predict potential public health risks. While the approach outlined here is not something that will be easily replicated by the general public, it can be replicated by health departments with some technical skills. Public health departments of large municipalities can build upon our methods and offer information to the public about the risks that have been identified by local citizens and aggregated via Yelp. There are biases inherent in the review data created by reviewers on Yelp, and by the proprietary algorithms applied by Yelp. This study is the first of its kind to use an epidemiologic approach to objectively evaluate this data source and measure its ability to predict substandard health code rating across different municipalities, and by using results of principal components analysis, inform inspectors about which aspects of reviewers’ observations drive predictions.

Our paper was reviewed by Dr. Tomás J. Aragón, Health Officer of the City and County of San Francisco and Director of the Population Health Division (PHD). By law, all counties in California have a physician health officer to exercise legal health authority, especially regarding communicable diseases. As PHD director, he directs all public health services including Environmental Health which conducts restaurant inspections, and Communicable Disease Control which conducts outbreak investigation. Dr. Aragón also teaches epidemiologic computing at the UC Berkeley School of Public Health, Division of Epidemiology.

According to Dr. Aragón this paper provides a novel data science method by using social media for predictive analytics of restaurant inspection scores (not outbreaks). With the economic boom and growth of the food establishments and vendors in San Francisco, and limited staff resources, this method will assist the SFDPH to prioritize which restaurants may require more frequent or intensive inspections. Without these predictive analytics, SFDPH can only rely on prior scores that reflect specific points in time, possibly more than a year old. The SFDPH has agreed to work with us in the future to deploy, evaluate, and improve this method for urban areas like San Francisco.

It is important to note that the system of assigning health code violations themselves is not a perfect one. Health inspectors only review the operations of restaurants a few times a year [6][29]. In contrast, Yelp reviewers in San Francisco, New York City, and other municipalities are creating hundreds to thousands of reviews for restaurants each and every year [23]. Yelp reviewers also analyze the safety of a restaurant in a far more direct way by actually consuming the food created by the restaurant. The real value of using social media data for public health surveillance is that not only does it catch elements that inspectors have already seen, but it also identifies what the inspectors have not yet seen, and may be unable to detect. One possible interpretation of prevalence prediction and lack of specificity would be that our model was actually able to detect cases that the SFDPH inspectors could not. Thus, we should not be overly conservative when judging the model’s sensitivity, AUC, and positive predictive value, because the standard against which the model is judged is far from perfect.

## Conclusion

Mining publicly-available, crowdsourced data to develop a surveillance method for tracking foodborne illness risk factors gives health inspectors an improved ability to identify restaurants with greater odds of low health code ratings and violations outside of the normal inspection window. Our approach uses available data and open source software. Our code for data extraction and analysis is available in public repositories and may be built upon to increase the breadth and transferability of our model. Additionally, tracking clusters of food safety compliance and foodborne illness-related keywords in large, crowdsourced data sets improves traditional surveillance methods without substantially increasing costs. This study serves as a step forward in illustrating how social media data may be used for the benefit of public health.

## Supporting Information

S1 Fig “Area Under the Receiver Operator Curve Across Page Rank” (AUC generated from simulated datasets with proportion of reviews ranging from 0% to 100%). (TIF)

S1 Table “Major Restaurant Health Violations in San Francisco” (List of all major health code violations used to form health code ratings in San Francisco). (PDF)
