# Supplementary figures and images for: Supplementing Public Health Inspection via Social Media
Source: PLoS One. 2016 Mar 29;11(3):e0152117. doi: 10.1371/journal.pone.0152117 (PMC4811425; doi:10.1371/journal.pone.0152117)

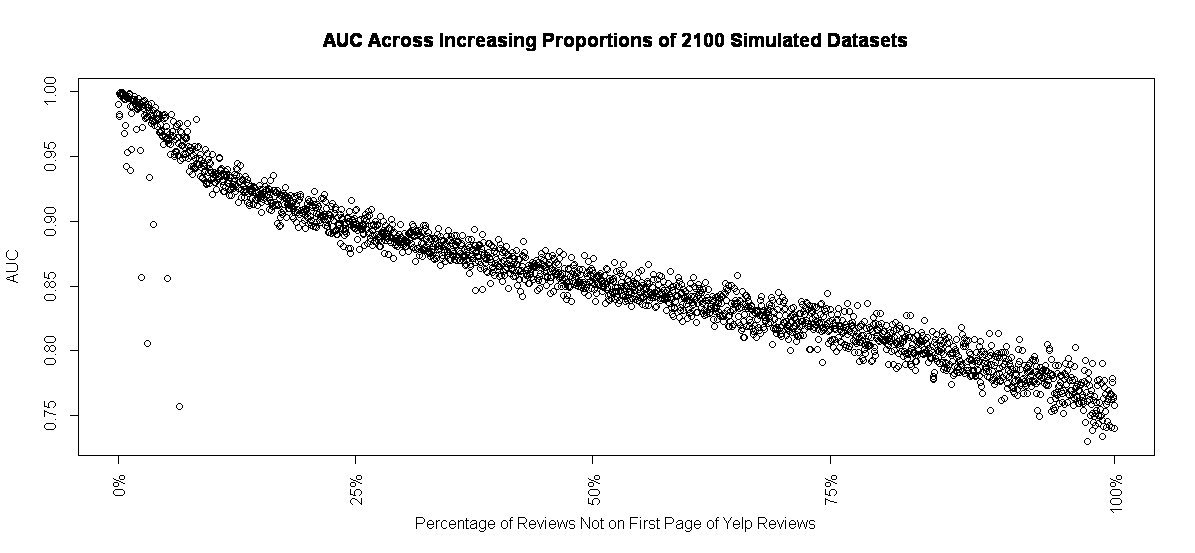

Supplement: S1 Fig — (TIF) [file pone.0152117.s001.tif]
