# Supplementary material for: Supplementing Public Health Inspection via Social Media
Source: PLoS One. 2016 Mar 29;11(3):e0152117. doi: 10.1371/journal.pone.0152117 (PMC4811425; doi:10.1371/journal.pone.0152117)
Supplement: S1 Table — (PDF) [file pone.0152117.s002.pdf]

Food Safety Program: Violation Terms

After conducting an inspection of your facility, the Environmental Health Inspector will calculate a score based on the violations observed. Different violations reflect different levels of risk to the public's health and safety. Major and minor violations are defined in the California Health and Safety Code (Part 7 - California Retail Food Code). Major violations pose an imminent public health danger and require immediate correction and/or closure of a facility. The following list defines the major violations that can occur during an inspection.

001 Communicable disease

Employees with a communicable disease shall be excluded from the food facility/preparation of food. Gloves shall be worn if an employee has cuts, sores, or rashes. No employee shall commit any act that may contaminate or adulterate food, food-contact surface, or utensils.

002 Hand washing

Employees are required to wash their hands before beginning work; before handling food/equipment/utensils; as often as necessary, during food preparation, to remove soil and contamination; when switching from working with raw to ready-to-eat foods, after touching body parts; using the toilet; or any time when contamination may occur.

003 Improper hot/cold holding temperatures

Two or more food items cannot be stored in separate containers/compartments or pooled eggs held in temperatures between 60˚F and 125˚F, with exceptions.

004 Time as a public health control

Written procedures for restricting time of food at unsafe temperatures to four hours is required; time control programs must be adhered to by staff.

005 Improper cooling methods

All potentially hazardous food shall be RAPIDLY cooled from 135˚F to 70˚F, within 2 hours, and then from 70˚F to 41˚F within 4 hours. Cooling shall be by one or more of the following methods: in shallow containers; separating food into smaller portions; adding ice as an ingredient; using an ice bath, stirring frequently; using rapid cooling equipment; or using containers that facilitate heat transfer.

006 Improper cooking time/temperatures

Food must be cooked to temperatures required by the California Health and Safety Code. Comminuted meat, or any food containing comminuted meat, shall be heated to 155˚F. Eggs and foods containing raw eggs, and single pieces of meat (including beef, veal, lamb, pork and approved game meat) shall be heated to 145˚F. Poultry, comminuted poultry, stuffed fish, and stuffed meat/poultry shall be heated to 165˚F.

007 Reheating

Any potentially hazardous food cooked, cooled and reheated for hot holding or serving shall be heated to 165˚F.

008 Food in poor condition; unsafe/adulterated

Any food is adulterated if it bears or contains any poisonous or deleterious substance that may render it impure or injurious to health.

009 Food contact surfaces not cleaned/sanitized

Food contact surfaces are to be cleaned and sanitized at an adequate frequency; a sanitizer is to be provided in the 3-compartment sink or final rinse of the dishwasher; and food contact surfaces must be washed, rinsed and sanitized before change in use.

010 Food obtained from an unapproved source

All food shall be obtained from an approved source.

011 Prohibited foods offered in licensed health care facilities/public and private schools

No raw or under-cooked food should be served at locations with highly susceptible populations. Examples of raw or under-cooked foods include unpasteurized juice, raw or under-cooked meat, etc.

012 No hot water/no water

Food preparation establishment shall have an adequate, protected, pressurized, potable supply of hot water (120˚F) and cold water shall be provided at all times.

013 Sewage/Wastewater disposal inoperative

A food facility cannot operate if sewage backs up into the facility, if a grease trap over flows or clogs, or when there are no operable toilets. All liquid waste must drain to an approved fully functioning sewage disposal system.

014 Rodents/Roaches/Flies/Other Animals

Each food facility shall be kept free of vermin.
